# Decoding the gut-adipose-ovary axis in polycystic ovary syndrome: from metabolic dysregulation to oncological risk modulation

**DOI:** 10.3389/fmicb.2026.1882127

**Published:** 2026-07-15

**Authors:** Lijuan Guo, Jialei Lu, Lanqing Liu, Minglei Zhang, Nan Yu, Le Lu, Tingting Yang, Jianbo Zhou, Bo Hou, Yuhan Chen, Yu Geng

**Affiliations:** 1 Department of Gynaecology and Obstetrics, Binhai County People’s Hospital, Yancheng, Jiangsu, China; 2 School of Pharmacy, Hangzhou Medical College, Hangzhou, Zhejiang, China; 3 Department of Acupuncture and Massage, The First People’s Hospital of Xuzhou, Xuzhou, Jiangsu, China; 4 Department of Oncology, Binhai County People’s Hospital, Yancheng, Jiangsu, China; 5 School of Public Health, Hangzhou Medical College, Hangzhou, Zhejiang, China; 6 Interventional Department, Binhai County People’s Hospital, Yancheng, Jiangsu, China

**Keywords:** gut microbiota, gut-adipose-ovary axis, oncological risk modulation, polycystic ovary syndrome, precision nutrition

## Abstract

Polycystic ovary syndrome (PCOS) is traditionally managed as a localized reproductive disorder, but emerging evidence redefines it as a systemic metabolic-endocrine continuum with profound long-term health implications. This review comprehensively synthesizes the pathogenic role of the nutrition-mediated “gut-adipose-ovary axis” in bridging PCOS-associated metabolic dysfunction with an elevated risk of gynecological malignancies. Aberrant nutritional intake acts as the primary environmental catalyst, driving gut microbiota dysbiosis and metabolic endotoxemia. These gut-derived signals provoke visceral adipose tissue dysfunction, initiating a self-reinforcing cascade of systemic insulin resistance, hyperandrogenism, and chronic low-grade inflammation. Crucially, the convergence of these systemic insults continuously remodels the localized ovarian microenvironment. By sustaining proliferative signaling, forcing metabolic reprogramming, and fostering immune evasion, these convergent insults may contribute to a permissive pre-neoplastic microenvironment in susceptible patients with PCOS. To dismantle this pathogenic network, we outline a multi-dimensional therapeutic framework that integrates precision nutrition, microbiome modulation, and targeted pharmacological agents. Ultimately, this systems-biology perspective mandates a paradigm shift in clinical practice: moving beyond empirical symptom palliation toward proactive, risk-stratified interventions that interrupt the disease continuum and reduce long-term oncological risk in appropriately stratified women with PCOS.

## 1 Introduction

Polycystic ovary syndrome (PCOS) is a complex and heterogeneous endocrine-metabolic disorder that affects 5 to 18% of reproductive-aged women worldwide, making it the most prevalent cause of anovulatory infertility and a significant contributor to long-term metabolic morbidity (Joham et al., 2022; Li et al., 2024). Clinically defined by the 2003 Rotterdam criteria—requiring at least two of hyperandrogenism, ovulatory dysfunction, and polycystic ovarian morphology—PCOS extends fundamentally beyond the reproductive axis. It is driven by multifactorial interactions among genetic susceptibility, neuroendocrine dysfunction, and environmental influences (Senthilkumar et al., 2025), manifesting as a cluster of metabolic disturbances that include central obesity, dyslipidemia, chronic low-grade inflammation, and insulin resistance (IR) (Siddiqui et al., 2022; Tanprasertsuk et al., 2021). Indeed, IR is a hallmark present in 30–95% of patients (Aswal et al., 2025), precipitating a vicious bidirectional cycle with adipose tissue dysfunction. In this state, an altered body fat distribution favors visceral adiposity, which secretes reduced levels of adiponectin alongside elevated pro-inflammatory cytokines, such as tumor necrosis factor-alpha (TNF-α) and interleukin-6 (IL-6) (Ni et al., 2025). This lipotoxic environment not only exacerbates systemic IR but actively stimulates ovarian androgen production, which reciprocally impairs adipocyte differentiation and lipolysis (Caretto et al., 2026; Ni et al., 2025). Over time, this unresolved metabolic dysregulation elevates the risk of severe sequelae, including type 2 diabetes mellitus (T2DM), cardiovascular disease, nonalcoholic fatty liver disease (NAFLD), and certain gynecological cancers (Wang and He, 2022; Wu et al., 2025). Despite decades of research, comprehensive insights into the systemic, multi-organ crosstalk propagating this phenotype remain elusive, often restricting clinical management to symptom palliation rather than targeted disease modification (Yadav et al., 2025a; Zhang et al., 2026). This broader systemic understanding has also prompted recent discussions regarding PCOS nomenclature, because the traditional term does not fully reflect the endocrine-metabolic and long-term cardiometabolic dimensions of the disorder.

Crucially, recent evidence highlights the gut microbiome as a key biological mediator contributing to this widespread metabolic and endocrine dysfunction (Zhu et al., 2025). Gut dysbiosis in PCOS—characterized by a pathologically altered Firmicutes/Bacteroidetes ratio (though directionality varies across clinical cohorts) and shifts in specific taxa like *Lactobacillus* and *Bifidobacterium*—is intrinsically pathogenic, as demonstrated by fecal microbiota transplantation (FMT) studies where microbiota from affected donors directly induces IR, hyperandrogenism, and ovarian dysfunction in healthy recipients (Fu et al., 2023; Yong et al., 2024). This microbial imbalance compromises intestinal barrier integrity, triggering metabolic endotoxemia and propagating the systemic inflammation that disrupts the gut-adipose axis (Patel, 2026). Furthermore, altered microbial metabolism of bile acids and short-chain fatty acids (SCFAs) directly impacts adipose functionality, notably contributing to the depletion of thermogenic brown adipose tissue (BAT) activity in experimental models, thereby entrenching metabolic inefficiency (Li-Hua and Bajinka, 2025). The convergence of this gut-adipose dysregulation exerts profound effects on the ovarian microenvironment. Compensatory hyperinsulinemia upregulates theca cell steroidogenic enzymes (e.g., CYP17A1, CYP11A1, HSD17B3) to drive luteinizing hormone (LH)-dependent androgen synthesis, while suppressing hepatic sex hormone-binding globulin (SHBG) to increase bioavailable testosterone (Yadav et al., 2025b). Concurrently, chronic inflammation drives granulosa cell apoptosis via pathways like IRE1α/XBP1 endoplasmic reticulum stress, enforcing the classic polycystic follicular arrest (Çıtar Dazıroğlu and Acar Tek, 2023; Zhang et al., 2021). Importantly, the sustained convergence of hyperandrogenism, insulin-like growth factor 1 (IGF-1) signaling, and oxidative stress may create conditions that favor the emergence of a pre-neoplastic microenvironment. This metabolically deranged environment promotes pro-proliferative and anti-apoptotic signaling (e.g., PI3K/Akt, MAPK), which, alongside gut-derived genotoxins, provides a mechanistic rationale for the epidemiological links between PCOS and gynecological malignancies, particularly endometrial cancer.

Because diet is the most rapidly modifiable determinant of the gut ecosystem, targeted nutritional intervention presents a compelling strategy to dismantle this pathogenic axis. Nutritional modifications—including the Mediterranean diet (MedDiet), low-glycemic index regimens, and the ingestion of specific dietary fibers or polyunsaturated fatty acids—have proven highly effective in restoring microbial diversity, enhancing BAT activity in preclinical models, and breaking the cycle of metabolic dysfunction (Kalupahana et al., 2020; Li S. et al., 2025; Patel, 2026). Meta-analyses confirm that targeted supplementation, such as probiotics or myo-inositol, significantly improves insulin sensitivity, reflected by improved Homeostatic Model Assessment for Insulin Resistance (HOMA-IR) scores, and reduces inflammatory markers like C-reactive protein (Wang et al., 2025). Building on these insights, this review provides a comprehensive mechanistic synthesis of how nutrition remodels the “gut-adipose-ovary” axis, thereby averting the transition from PCOS-associated metabolic dysfunction to a pre-neoplastic state. We systematically explore evidence across five critical domains: (1) the role of dietary patterns in dictating gut microbiota architecture and subsequent metabolic states; (2) the molecular signaling cascades linking gut-derived metabolites to IR and adipose dysfunction; (3) the downstream impact of this deranged milieu on ovarian folliculogenesis and steroidogenesis; (4) the mechanistic and epidemiological connections between PCOS and gynecological cancer risk; and (5) the therapeutic efficacy of targeted nutritional and nutrigenomic approaches—including bioactive phytochemicals like curcumin, mangiferin, and myricetin—in restoring axis homeostasis. By mapping these multidimensional interactions, we propose an integrated model of disease progression and highlight actionable dietary strategies capable of mitigating both the endocrine-metabolic and early cancer-related consequences of PCOS.

## 2 The concept and composition basis of the gut-adipose-ovary axis

### 2.1 Core components and functional interconnectivity of the axis

The gut-adipose-ovary axis functions as a sophisticated, multi-directional communication network that integrates metabolic, endocrine, and immune signaling to maintain female reproductive homeostasis. This network is anchored by three primary biological structures, beginning with the gut, which comprises the intestinal epithelial barrier and its resident microbiota. The barrier selectively facilitates nutrient absorption while restricting the systemic translocation of deleterious luminal contents, such as bacterial endotoxins (Ilias et al., 2021). The microbial ecosystem generates critical bioactive metabolites, predominantly SCFAs (including butyrate, propionate, and acetate), secondary bile acids, and tryptophan derivatives like indole-3-propionic acid (IPA) (Li Z. et al., 2025). In metabolic disorders like PCOS, microbial dysbiosis compromises barrier integrity—a phenomenon clinically termed “leaky gut”—permitting Gram-negative lipopolysaccharides (LPS) to infiltrate the circulation and precipitate chronic low-grade inflammation (Senthilkumar and Arumugam, 2025; Zhang et al., 2024). The second pillar is adipose tissue, specifically visceral adipose tissue (VAT). Operating far beyond a passive energy reservoir, VAT acts as a highly active endocrine organ, secreting a distinct profile of adipokines (e.g., leptin, adiponectin, resistin) alongside pro-inflammatory cytokines such as TNF-α and IL-6 (Ni et al., 2025). During obesity or PCOS, hypertrophic VAT frequently becomes infiltrated with M1-polarized macrophages (Feng et al., 2023), disrupting adipokine homeostasis. This localized inflammation drives leptin resistance, diminishes insulin-sensitizing adiponectin, and amplifies pro-inflammatory signaling (Ferreira et al., 2026). Concurrently, the impairment of BAT activity and the blunted “browning” of white adipose tissue (WAT)—processes critical for thermogenesis via uncoupling protein 1 (Ucp1)—severely restrict energy expenditure and metabolic flexibility (Qi et al., 2020). The third component is the ovary, a dynamic organ whose primary functions (folliculogenesis, steroidogenesis, and ovulation) are exquisitely sensitive to systemic metabolic cues. While governed locally by gonadotropins, the ovarian microenvironment is heavily perturbed by systemic aberrations, as compensatory hyperinsulinemia stemming from IR directly forces theca cell androgen hypersecretion (Hernández-Jiménez et al., 2022; Huang et al., 2025). Furthermore, elevated circulating LPS binds to Toll-like receptor 4 (TLR4) on ovarian immune and somatic cells, initiating local inflammatory cascades that derail steroidogenesis and accelerate follicular atresia.

The physiological integrity of this axis relies entirely on the robust functional interconnectivity among its components, establishing a bi-directional signaling network. Microbial metabolites act as central endocrine messengers within this triad, as illustrated by systemically circulating SCFAs that activate G-protein-coupled receptor 43 (GPR43) on peri-ovarian gonadal white adipose tissue (gWAT) (Yang X. et al., 2025). This localized activation stimulates leptin secretion, which subsequently signals the ovary to suppress granulosa cell apoptosis and promote follicular maturation (Xu et al., 2025). Similarly, the gut-derived derivative IPA mitigates PCOS pathogenesis by engaging the aryl hydrocarbon receptor (AhR) pathway, thereby dampening systemic inflammation and restoring ovarian morphology (Li Z. et al., 2025). Beyond microbial metabolites, classical hormones and cytokines serve as vital conduits. Adipose-derived leptin directly modulates the hypothalamic–pituitary-ovarian axis, whereas inflammatory mediators like TNF-α independently impair ovarian steroidogenesis and exacerbate peripheral IR (Zhang H. et al., 2023). Importantly, this crosstalk is inherently reciprocal, as ovarian hormones potently shape the gut microbiome. Estrogen deficiency precipitates severe microbial dysbiosis—marked by the depletion of beneficial taxa such as *Akkermansia muciniphila*—which in turn amplifies downstream metabolic disturbances (Rishabh et al., 2024). Consequently, pathogenic alterations originating in any single node of the gut-adipose-ovary axis inevitably propagate through the entire network, entrenching the vicious cycle of metabolic and reproductive dysfunction characteristic of PCOS.

### 2.2 Nutritional intake as the central modulator of the axis

As the primary environmental driver, nutritional intake fundamentally dictates the homeostatic balance or pathological drift of the gut-adipose-ovary axis. The chronic overconsumption of saturated fatty acids and refined carbohydrates acts as a dominant catalyst for axis derangement. A high-fat diet (HFD), operating synergistically with preexisting hyperandrogenism, induces adipocyte hypertrophy, elevates circulating androgens, and disrupts the estrous cycle (Xu et al., 2026). This obesogenic environment fosters a self-perpetuating cycle wherein inflamed, dysfunctional VAT exacerbates systemic IR and stimulates further ovarian androgenesis, ultimately impairing glucose tolerance and accelerating fat depot expansion (Ferreira et al., 2026; Ni et al., 2025). Additionally, excessive dietary intake of branched-chain amino acids (BCAAs) frequently drives IR by altering microbiota composition and aberrantly activating the mTORC1 signaling pathway, thereby upregulating pro-inflammatory gene expression (Jia and Li, 2024; Tanase et al., 2024). Conversely, targeted nutritional interventions exert profound protective effects, primarily by remodeling the microbial ecosystem. Dietary fibers enhance SCFA production, yielding widespread systemic benefits. While gut-derived butyrate generally ameliorates IR and reproductive dysfunction—often facilitating gWAT-mediated leptin signaling via GPR43—its therapeutic efficacy is highly context-dependent. In models with pre-established obesity and hyperandrogenism, exogenous sodium butyrate paradoxically exacerbates adipose inflammation, highlighting the necessity of context-specific nutritional strategies (Ferreira et al., 2026). Similarly, polyunsaturated fatty acids (PUFAs), particularly omega-3 s, directly attenuate ovarian inflammation (e.g., IL-1β, TNF-α) while enriching beneficial gut taxa, thereby improving subcutaneous adipose morphology and dampening pathological thermogenic markers (Zhang H. et al., 2023). Nutritional signals also engage systemic metabolic pathways, with the nutrient-responsive protein Orosomucoid 2 (ORM2) upregulating Ucp1 expression in WAT to significantly improve ovarian phenotypes through adipose browning (Xu et al., 2026).

Beyond acute metabolic modulation, prolonged dietary patterns exert enduring, plastic effects via epigenetic reprogramming. Sustained adherence to anti-inflammatory diets can imprint a favorable epigenetic landscape, whereas chronic HFD exposure establishes a metabolic memory characterized by entrenched IR. This physiological plasticity underscores the efficacy of comprehensive dietary regimens, such as the ketogenic diet (KD), which couples profound metabolic correction—lowering triacylglycerols and restoring insulin sensitivity—with immune modulation, potentially by attenuating nuclear factor kappa-light-chain-enhancer of activated B cells (NF-κB) signaling (Yang W. et al., 2025). Moreover, diet-induced shifts in the hormonal milieu dictate microbial architecture, as estrogen fluctuations dramatically alter the ratio of beneficial microbes (*Lactobacillus* spp., *Faecalibacterium prausnitzii*) to opportunistic pathogens (*Clostridium difficile*), reinforcing the interconnected feedback loops of the axis (Farhat et al., 2023; Rishabh et al., 2024). Ultimately, nutrition transcends mere caloric provision, functioning as a potent, chronic modulator that shapes the structural and functional resilience of the gut-adipose-ovary axis through metabolite generation, endocrine signaling, and epigenetic reprogramming.

## 3 Axis mechanisms of nutritional dysregulation driving PCOS metabolic phenotypes

**Figure 1:**
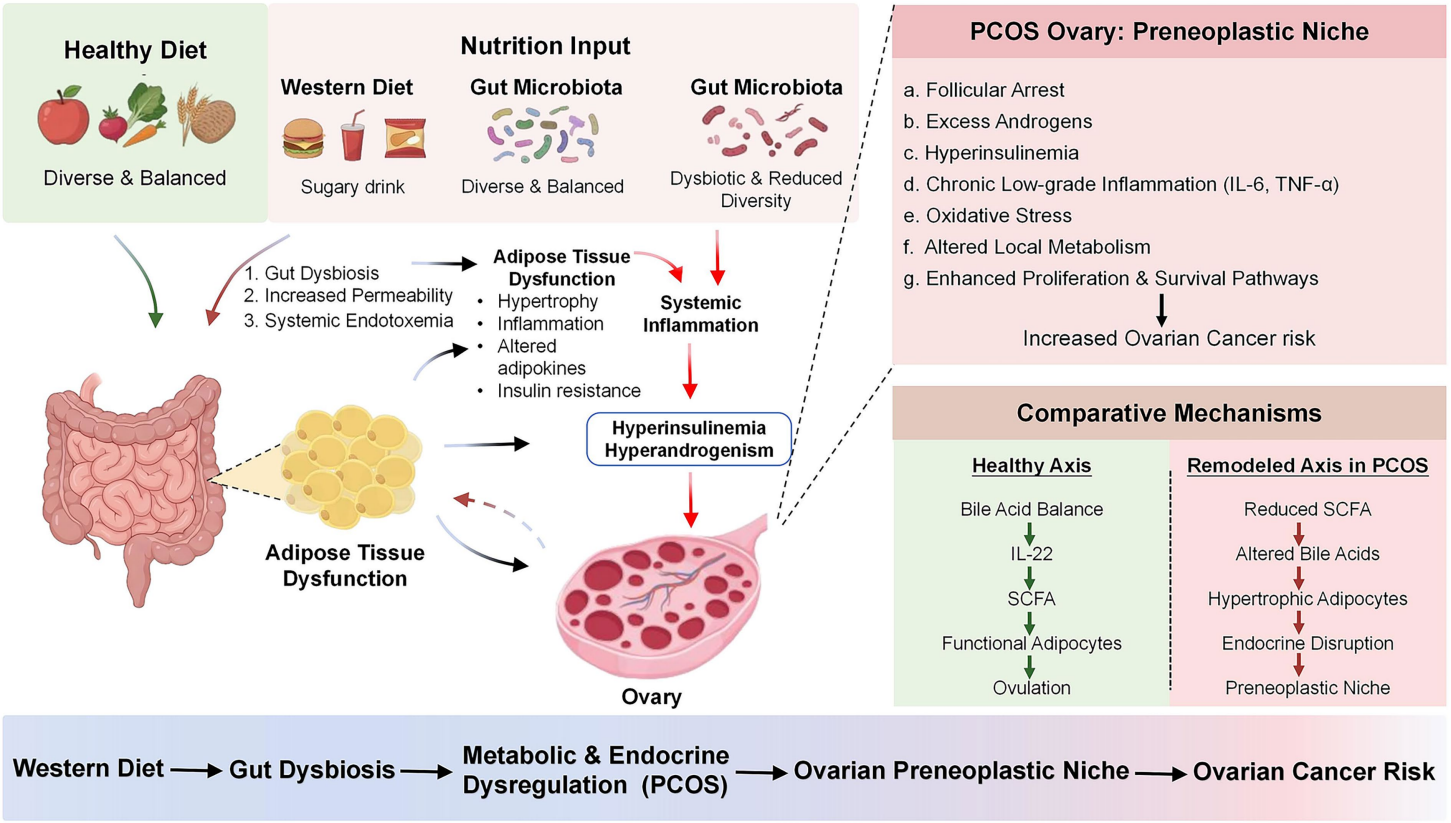
Pathological mechanisms of nutrition-mediated gut-adipose-ovary axis remodeling in PCOS and the potential formation of a permissive ovarian preneoplastic microenvironment. This diagram illustrates the differential impacts of varying dietary patterns on the “gut-adipose-ovary axis” and their potential contributing roles in the metabolic dysregulation and long-term complications of PCOS. In contrast to a healthy diet, a Western diet leads to gut microbiota dysbiosis, which triggers increased intestinal permeability and systemic endotoxemia (LPS). These gut-derived pathological stimuli further induce visceral adipose tissue (VAT) dysfunction, characterized by adipocyte hypertrophy, local inflammation, and insulin resistance, ultimately evolving into systemic inflammation, hyperinsulinemia, and hyperandrogenism. At the local reproductive level, these systemic metabolic and endocrine derangements collectively remodel the microenvironment, thereby favoring the development of a permissive “Ovarian Preneoplastic Niche” rather than indicating inevitable malignant transformation. The pathological features of this niche include: (a) follicular arrest, (b) excess androgens, (c) hyperinsulinemia, (d) chronic low-grade inflammation (IL-6, TNF-α), (e) oxidative stress, (f) altered local metabolism, and (g) enhanced proliferation and survival pathways, which may contribute to increased long-term cancer-related risk in susceptible PCOS phenotypes. The comparison module (bottom right) summarizes the mechanistic differences between the healthy axis (Bile Acid balance, IL-22, SCFA, functional adipocytes, and ovulation) and the remodeled axis in PCOS (reduced SCFA, altered Bile Acids, hypertrophic adipocytes, endocrine disruption, and the preneoplastic niche).

As conceptually illustrated in the left and central panels of Figure 1, nutritional dysregulation initiates a sequential cascade from gut microbiota dysbiosis and barrier impairment to adipose tissue dysfunction, systemic inflammation, insulin resistance, and ovarian endocrine disruption. The following subsections unpack each component of this mechanistic flow.

### 3.1 Gut microbiota dysbiosis and metabolic endotoxemia

Diets rich in fats and refined sugars serve as primary catalysts for gut microbiota dysbiosis, precipitating a steep decline in microbial diversity and fundamentally shifting community architecture. This nutritional pattern enriches conditionally pathogenic taxa, notably within the Bacteroidetes phylum, while simultaneously depleting beneficial, SCFA-producing populations such as *Akkermansia muciniphila* (Li et al., 2021; Qiao et al., 2022). This ecological disruption represents a critical pathogenic event that severely compromises intestinal barrier integrity. Specifically, the altered microbial milieu suppresses the expression and structural organization of essential epithelial tight junction proteins—including zonula occludens-1 (ZO-1), Occludin, and Claudin-1—culminating in pathological intestinal permeability, clinically recognized as “leaky gut” (Cho et al., 2026; Gasmi et al., 2021; Wang S. et al., 2026). The deterioration of this physical barrier facilitates the systemic translocation of bacterial endotoxins, predominantly LPS from the cell walls of Gram-negative bacteria, escaping the gut lumen into the portal circulation. This phenomenon establishes a state of metabolic endotoxemia (Ganamurali and Sabarathinam, 2026; Park et al., 2024). Upon entering systemic circulation, LPS binds to and activates TLR4 on both immune and metabolic cells localized within the liver and adipose depots (Abbasi et al, 2025; Bian et al., 2022; Ning et al., 2025). Subsequent TLR4-mediated signaling provokes a robust inflammatory cascade, characterized by the unabated secretion of cytokines such as TNF-α and IL-6. This initiates a chronic, low-grade inflammatory state that contributes to systemic IR (Kangwan et al., 2021). Consequently, this mechanistic sequence—from diet-induced dysbiosis and barrier failure to endotoxin translocation and TLR4-driven inflammation—constitutes a foundational axis linking nutritional excess to the core metabolic derangements of PCOS, actively enforcing an inflammatory environment that persistently dismantles glucose homeostasis and insulin signaling (Del Cornò et al., 2026; Rusman et al., 2025; Wang et al., 2024).

### 3.2 Adipose tissue dysfunction and adipokine imbalance

Nutritional excess, particularly prolonged exposure to a HFD, forces the pathological expansion of adipose tissue, characterized by a preferential and detrimental accumulation of VAT. This rapid adipocyte hypertrophy frequently outpaces local angiogenesis, subjecting the tissue to severe hypoxia and endoplasmic reticulum stress (Newman et al., 2023; Perdicaro et al., 2026). In response, these stressed and metabolically dysfunctional adipocytes secrete chemokines that actively recruit circulating monocytes. Upon infiltrating the adipose stroma, these immune cells polarize into the pro-inflammatory M1 macrophage phenotype (La Barbera et al., 2022). This localized macrophage activation establishes a self-amplifying inflammatory loop, wherein M1 macrophages continuously secrete copious volumes of inflammatory cytokines, notably TNF-α and IL-6, which progressively exacerbate both local and systemic IR (Feng et al., 2021; Kangwan et al., 2021). Concurrently, the intrinsic endocrine function of adipose tissue undergoes profound dysregulation. While leptin, an adipokine responsible for appetite suppression and energy expenditure, is hypersecreted, peripheral target tissues rapidly develop leptin resistance, thereby neutralizing its satiety signals and enforcing a sustained positive energy balance (Blanco, 2020). Conversely, the secretion of adiponectin—a potent insulin-sensitizing and anti-inflammatory mediator—is severely blunted (Chung et al., 2020). This inverted adipokine ratio (hyperleptinemia coupled with hypoadiponectinemia) represents the molecular hallmark of dysfunctional adipose tissue in obesity. Crucially, the repercussions of this lipotoxic state extend far beyond peripheral metabolism, directly and indirectly disrupting the hypothalamic–pituitary-ovarian (HPO) axis. Hyperleptinemia pathologically accelerates gonadotropin-releasing hormone (GnRH) pulsatility and alters intraovarian steroidogenesis (Wołodko et al., 2025), while the concurrent deficit in adiponectin removes a critical inhibitory brake on ovarian androgen biosynthesis. Thus, dysfunctional VAT operates dually as a continuous generator of systemic inflammation and a potent disruptor of endocrine signaling, cultivating a physiological landscape that actively drives the hyperandrogenism central to PCOS pathogenesis.

### 3.3 Metabolic-inflammatory remodeling of the ovarian local microenvironment

The ovarian microenvironment does not exist in isolation but rather functions as a highly vulnerable target for the systemic metabolic disturbances, inflammatory mediators, and hormonal aberrations originating from the dysregulated gut and VAT. The convergent impact of systemic inflammation—driven by elevated TNF-α and IL-6—and compensatory hyperinsulinemia profoundly remodels ovarian physiology (Dobre et al., 2025; Feng et al., 2021). Within the ovary, insulin and IGF-1 exert synergistic, stimulatory effects on theca cells. By binding to their respective receptors, they upregulate downstream intracellular signaling cascades that force the overexpression of essential steroidogenic enzymes, most notably CYP17A1 (Higuchi et al., 2020; Rawan et al., 2023). Because this enzyme governs a rate-limiting step in androgen biosynthesis, its hyperactivation directly fuels the continuous overproduction of ovarian testosterone, clinically manifesting as the cardinal hyperandrogenism of PCOS. Furthermore, the ovary itself transitions into an epicenter of localized low-grade inflammation. Somatic ovarian compartments, including granulosa cells, begin synthesizing their own inflammatory cytokines in response to systemic endotoxemia and localized oxidative stress, thereby creating and maintaining a localized inflammatory environment within the ovary (Orisaka et al., 2023). This hostile microenvironment severely disrupts the delicate autocrine and paracrine signaling networks requisite for healthy folliculogenesis. It impairs granulosa cell proliferation, skews the equilibrium between pro- and anti-apoptotic regulatory factors, and ultimately stalls ovulation. The synergistic assault of hyperinsulinemia-induced hyperandrogenism and localized inflammation fundamentally prevents dominant follicle selection and maturation. This arrest leads directly to chronic anovulation and the morphological accumulation of small, atretic antral follicles—the ultrasound-detectable polycystic ovarian morphology (Cagnacci et al., 2025; Zhang Y. et al., 2023). Ultimately, this pathogenic triad of systemic inflammation, IR-driven hyperinsulinemia, and a localized inflammatory intraovarian state comprehensively remodels the ovarian niche, mechanistically locking the patient into the metabolic and reproductive dysfunctions defining PCOS. The systemic and localized cascade, from aberrant nutritional input to the establishment of the pre-neoplastic niche, is visually integrated in Figure 1. As illustrated, a Western-style diet acts as a major environmental catalyst, driving gut microbiota dysbiosis and metabolic endotoxemia, which subsequently trigger a self-reinforcing loop of visceral fat dysfunction and systemic hyperandrogenism. This axis remodeling ultimately culminates in the formation of a pro-tumorigenic ovarian-endometrial environment.

## 4 Potential evolution from PCOS metabolic dysregulation to a premalignant ovarian–endometrial microenvironment

Current evidence supports a strong association between PCOS and endometrial cancer risk, whereas the relationship between PCOS and ovarian cancer remains more heterogeneous and requires further longitudinal validation. Therefore, the oncological component of the gut–adipose–ovary axis should be interpreted as a mechanistic and translational framework, rather than as evidence of inevitable malignant progression. As summarized in the right panel of Figure 1, systemic metabolic-endocrine dysregulation may progressively remodel the local ovarian microenvironment. The following section further extends this concept to the ovarian–endometrial interface, where chronic anovulation, progesterone deficiency, hyperinsulinemia, hyperandrogenism, inflammation, and stromal remodeling may jointly contribute to a cancer-related microenvironment in susceptible PCOS phenotypes.

### 4.1 Definition and characteristics of the precancerous niche

The “precancerous niche” is formally defined as a localized tissue microenvironment that undergoes sustained, pro-oncogenic alterations prior to the overt manifestation of malignancy (Gao et al., 2026). Far from acting as a static physiological backdrop, this is a dynamic, co-evolutionary ecosystem wherein mutant cells and their surrounding stroma engage in reciprocal selection. This intricate crosstalk ultimately determines whether a nascent neoplastic lesion will be eradicated, remain indolent, or progress to invasive disease (Gao et al., 2026). This conceptual framework fundamentally reframes tumor initiation—shifting the paradigm from a purely stochastic accumulation of somatic mutations to a microenvironmental selection process dictated by ecological gatekeeping (Jiang et al., 2024). Crucially, the defining features of this niche mirror the classical hallmarks of cancer, yet they are firmly established during the pre-invasive stage. These encompass sustained proliferative signaling, evasion of growth suppression, resistance to apoptosis, genomic instability, induction of angiogenesis, tumor-promoting inflammation, and metabolic reprogramming.

In the specific context of PCOS, the chronic metabolic-inflammatory state perpetuated by the dysregulated gut-adipose-ovary axis cultivates a systemic and localized reproductive niche that embodies pre-malignant features. Local ovarian inflammation and oxidative stress may increase tissue susceptibility to cellular transformation. At the same time, chronic anovulation and progesterone deficiency caused by axis dysfunction can place sustained proliferative pressure on the endometrial lining. For this reason, the cancer-related microenvironment in PCOS should be considered as an integrated ovarian–endometrial unit rather than as an isolated ovarian process. Within this unit, systemic hyperinsulinemia and hyperandrogenism interact with local stromal and inflammatory remodeling to promote early cancer-associated features. Importantly, early neoplastic cells are far from passive residents, as they actively instruct their stroma to engineer a supportive niche. For instance, in the upper gastrointestinal tract, nascent squamous tumors exploit stress responses to instruct the underlying mesenchyme to assemble a fibronectin-rich stromal scaffold. This remodeled matrix alone is sufficient to confer tumorigenic properties onto adjacent normal epithelial cells, as validated by functional 3D culture assays and *in vivo* grafting experiments (Skrupskelyte et al., 2026). This demonstrates that the precancerous niche can serve as the dominant driver of lesion persistence and progression, even in the absolute absence of additional epithelial mutations. Similarly, in Li-Fraumeni syndrome, while the germline TP53 mutation imparts systemic susceptibility, the localized precancerous niche remains the primary, targetable driver for chemoprophylactic intervention (Amador-Gómez et al., 2024).

The architectural maturation of this niche is further coordinated by “leader cells” that emerge during late precancerous stages. In esophageal squamous cell carcinoma, for example, these specialized cells actively breach the epithelial-stromal boundary and recruit normal fibroblasts via JAG1-NOTCH1 signaling, transdifferentiating them into cancer-associated fibroblasts (CAFs). This process establishes a protective “CAF-Epi” niche that effectively shields the nascent tumor from immune surveillance, serving as a critical harbinger of disease progression and poor clinical outcomes (Chang et al., 2025). Even in BRCA1 mutation carriers, the pre-neoplastic stroma undergoes fundamental alterations long before overt malignancy, a process in which pre-cancer-associated fibroblasts (pre-CAFs) secrete pro-proliferative mediators—such as matrix metalloproteinase-3 (MMP3)—that function in trans to aggressively drive epithelial proliferation and tumorigenesis (Nee et al., 2023). Collectively, these paradigms emphasize that the precancerous niche is an active, instructive microenvironment co-opted by early malignant cells to ensure survival, prioritizing the role of microenvironmental selection over mere mutation accumulation (Gao et al., 2026). The inflammatory axis is particularly indispensable here, as a cytokine-rich niche (e.g., driven by IL-6) represents a highly conserved feature across multiple malignancies, notably driving the expansion of early progenitor populations, such as alveolar progenitors in lung adenocarcinoma (Sasaki et al., 2026; Xiang et al., 2025).

### 4.2 Specific mechanisms of axis dysregulation driving the precancerous niche

Dysregulation of the gut–adipose–ovary axis in PCOS may promote a permissive pre-neoplastic microenvironment through several interconnected mechanisms that resemble early cancer-associated hallmarks. Sustained proliferative signaling operates as the structural cornerstone of the PCOS-associated precancerous niche. Pathologically elevated levels of insulin and IGF-1, resulting from systemic IR and hyperandrogenism, robustly activate the PI3K/Akt/mTOR signaling cascade within ovarian epithelial cells (Leslie et al., 2022). This hyperactivated pathway delivers a potent mitogenic signal that overrides physiological growth checkpoints, thereby driving unchecked cellular proliferation. Because this unyielding proliferative drive is a defining prerequisite for early tumorigenesis, the hyperinsulinemic state directly anchors the “sustained proliferative signaling” hallmark within the ovarian microenvironment.

Tumor-promoting inflammation functions as a secondary, yet equally critical, mechanistic pillar. The chronic systemic and localized ovarian inflammation—characterized by elevated TNF-α and IL-6—engineers a profoundly pro-tumorigenic milieu (Cui et al., 2022). This persistent inflammatory signaling triggers the overproduction of reactive oxygen species (ROS) and reactive nitrogen species (RNS), which actively inflict oxidative DNA damage and accelerate the accumulation of genomic mutations (Santhakumar et al., 2020). Furthermore, these inflammatory mediators trigger the activation of oncogenic transcription factors, including signal transducer and activator of transcription 3 (STAT3) and NF-κB, in adjacent epithelial cells. This activation reinforces a robust pro-survival and pro-proliferative transcriptional program (Cui et al., 2022). The concurrent activation of the IL-1β pathway further highlights the role of the inflammasome in sculpting an inflammatory niche that actively fuels early oncogenic mutations (Nasrollahzadeh et al., 2020; Wilson et al., 2023). Within the context of PCOS, this establishes a perilous feed-forward loop where chronic inflammation and genomic instability perpetually reinforce one another.

Metabolic reprogramming, classically known as the Warburg effect, is actively integrated into the PCOS-driven precancerous niche. Despite adequate oxygen availability, the aberrant hyperactivation of the PI3K/Akt pathway, combined with localized hypoxic signaling (mediated via the stabilization of hypoxia-inducible factor 1-alpha, HIF-1α), forces ovarian epithelial cells to shunt glucose metabolism toward aerobic glycolysis (Eubank et al., 2024). This metabolic switch rapidly supplies proliferating cells with essential biosynthetic precursors—such as nucleotides, amino acids, and lipids—required for sustained clonal expansion. Simultaneously, this glycolytic shift generates increased levels of lactic acid as a byproduct. The resulting acidification of the extracellular microenvironment aggressively facilitates tumor progression by remodeling the extracellular matrix and actively suppressing anti-tumor immune responses. As an early event in carcinogenesis, this transition from oxidative phosphorylation to glycolysis is a direct, downstream consequence of the axis-driven hyperinsulinemic and hyperandrogenic state.

Immune surveillance escape constitutes the critical final step enabling transformed cells to survive and proliferate unabated. The chronic inflammatory milieu established by the dysregulated axis actively recruits and expands potent immunosuppressive leukocyte populations, notably regulatory T cells (Tregs) and myeloid-derived suppressor cells (MDSCs) (Han et al., 2020). Working in concert with tumor-associated macrophages (TAMs)—which are frequently polarized toward a pro-tumorigenic M2-like phenotype—these populations construct a formidable immunosuppressive barrier (Poh and Ernst, 2021). TAMs secrete paracrine factors that preserve tumor stemness and paralyze cytotoxic T-cell function, while MDSCs independently suppress T-cell proliferation (Goto et al., 2025). Crucially, rather than being incidental, this immune evasion represents a highly orchestrated process driven by early neoplastic cells and the surrounding dysregulated stroma to shield aberrant cells from immune clearance, granting them the temporal window required to acquire advanced oncogenic traits (Goto et al., 2025). Ultimately, the formation of this immune-excluded “cold” microenvironment is an important feature of the PCOS-driven precancerous niche and a potential facilitator of malignant transformation. As summarized in Table 1, the continuous progression from metabolic dysregulation to the precancerous niche involves a complex, multi-organ cascade. This table systematically outlines the key pathological alterations at each critical node—from the gut microbiota and intestinal barrier to visceral adipose tissue and the ovarian-endometrial axis—and highlights their primary downstream consequences in facilitating early oncogenesis.

**Table 1:** Pathophysiological mechanisms of the gut-adipose-ovary axis and potential cancer-related microenvironmental remodeling in PCOS.

Target node/microenvironment	Key pathological alterations	Primary downstream consequences	References
Gut microbiota and intestinal barrier	Altered Firmicutes/Bacteroidetes ratio and depletion of SCFA-producing taxa (e.g., *Akkermansia muciniphila*). Downregulation of tight junction proteins (ZO-1, Occludin)	Compromised barrier integrity (“leaky gut”) facilitating the systemic translocation of Gram-negative lipopolysaccharides (LPS) and inducing metabolic endotoxemia	Li et al. (2021), Cho et al. (2026), Park et al. (2024)
Visceral adipose tissue (VAT)	Rapid adipocyte hypertrophy and hypoxia triggering endoplasmic reticulum stress. Infiltration of M1-polarized macrophages and dysregulated adipokine secretion (high leptin, low adiponectin)	Sustained release of pro-inflammatory cytokines (TNF-α, IL-6), exacerbation of systemic insulin resistance (IR), and impairment of thermogenic brown adipose tissue (BAT) activity	Qi et al. (2020), Perdicaro et al. (2026), La Barbera et al. (2022), Feng et al. (2021)
Ovarian-endometrial axis	Synergistic action of insulin/IGF-1 on theca cells overexpressing steroidogenic enzymes (e.g., CYP17A1). Localized accumulation of pro-inflammatory cytokines causing granulosa cell apoptosis	Continuous ovarian testosterone overproduction (hyperandrogenism) and chronic anovulation, resulting in progesterone deficiency that may impose sustained proliferative pressure on the endometrium	Higuchi et al. (2020), Orisaka et al. (2023), Zhang Y. et al. (2023)
The precancerous niche	Hyperactivation of the PI3K/Akt/mTOR signaling cascade and metabolic reprogramming toward aerobic glycolysis (Warburg effect). Overproduction of ROS/RNS and recruitment of immunosuppressive cells (Tregs, MDSCs)	Unchecked cellular proliferation, oxidative DNA damage, immune surveillance escape, and establishment of a highly permissive microenvironment for cellular transformation	Leslie et al. (2022), Santhakumar et al. (2020), Eubank et al. (2024), Han et al. (2020)

## 5 Key molecular bridges and signaling pathway integration

### 5.1 The role of the bile acid metabolic axis

The bile acid (BA) metabolic axis operates as a critical chemical signaling conduit linking the gut microbiota to distal metabolic organs, including the liver, adipose tissue, and ovaries, thereby orchestrating the pathophysiology of metabolic dysregulation in PCOS (Zhang et al., 2022). The intestinal microbial ecosystem is indispensable for the biotransformation of hepatic-synthesized primary BAs into secondary BAs—such as deoxycholic acid (DCA) and lithocholic acid (LCA)—via deconjugation and dehydroxylation reactions (Qian et al., 2026). These metabolites function as distinct endocrine signals mediated through two primary receptors (Fiorucci et al., 2022). Primary BAs preferentially activate the nuclear farnesoid X receptor (FXR), which is predominantly expressed in the liver and intestine to govern the enterohepatic circulation of BAs and modulate core metabolic pathways, including gluconeogenesis and lipogenesis (Chiang et al., 2020). Conversely, microbial-derived secondary BAs (such as DCA and LCA) serve as potent agonists for the membrane-bound G protein-coupled bile acid receptor 1 (TGR5), which is widely distributed across brown and white adipose tissue, skeletal muscle, and immune cells to drive systemic energy expenditure and exert potent anti-inflammatory effects (Qi et al., 2022). In the metabolically dysregulated state of PCOS, this axis is severely disrupted, characterized by diminished total BA pools and a pathological shift in the primary-to-secondary BA ratio (Ravat et al., 2024). This dysbiosis-induced alteration actively impairs FXR/TGR5 signaling, thereby derailing hepatic glucose and lipid homeostasis. Specifically, aberrant hepatic FXR activation exacerbates IR and dyslipidemia, while the parallel failure of TGR5 signaling in adipose tissue blunts thermogenesis, enforcing an obese phenotype (Chiang and Ferrell, 2022; Mustonen et al., 2021). Beyond peripheral metabolism, the BA-FXR/TGR5 axis directly penetrates the ovarian microenvironment to regulate steroidogenesis and localized inflammation. Pathologically elevated levels of specific BAs, such as glycochenodeoxycholic acid, tightly correlate with hyperandrogenemia in PCOS, and excessive BA accumulation induces granulosa cell apoptosis via endoplasmic reticulum stress (Liu and Zhang, 2026). Conversely, the administration of protective BAs, like tauroursodeoxycholic acid (TUDCA), mitigates this cellular stress and restores oocyte quality (Freitas et al., 2023).

Consequently, the gut microbiota-BA-ovary axis represents a primary vector through which intestinal dysbiosis propagates metabolic and reproductive failure. Therapeutic interventions designed to remodel the microbial ecosystem—including dietary fiber supplementation, probiotics, and bariatric surgery—successfully restore BA homeostasis and attenuate PCOS phenotypes. For instance, oligofructose supplementation elevates circulating hyodeoxycholic acid (HDCA), which enhances ovarian aromatization and suppresses hyperandrogenism in murine models (Wang Y. et al., 2026). Similarly, physical exercise enriches secondary BAs like DCA, which subsequently activates hepatic FXR to suppress gluconeogenesis and improve insulin sensitivity (Li Q. et al., 2025). Biliopancreatic diversion with duodenal switch (BPD/DS) mechanically drives a dramatic surge in serum BAs, correlating directly with the amelioration of IR and reproductive disorders (Li et al., 2026). Ultimately, the BA metabolic axis functions as a master integrator of host-microbiota crosstalk, positioning its targeted regulation as a highly promising therapeutic strategy for restoring physiological homeostasis in PCOS.

### 5.2 The “double-edged sword” effect of short-chain fatty acids

SCFAs—primarily acetate, propionate, and butyrate—are the dominant end-products of microbial dietary fiber fermentation and function as fundamental regulators of host metabolism and immunity (Babu et al., 2024). However, their physiological impact is distinctly context-dependent, manifesting as a “double-edged sword.” While universally recognized for their metabolic protection, aberrant SCFA signaling within specific microenvironments can paradoxically facilitate pathological, and even oncogenic, processes.

In the metabolically deranged state of PCOS, inadequate dietary fiber intake starves fermentative taxa, precipitating a profound systemic SCFA deficiency (Song et al., 2025). This depletion critically dismantles multiple homeostatic mechanisms. First, the potent anti-inflammatory effects of SCFAs are abolished. Under normal physiological conditions, butyrate acts as a natural histone deacetylase (HDAC) inhibitor, suppressing pro-inflammatory cytokine transcription to prevent the chronic low-grade inflammation typical of PCOS (Li Q. et al., 2025). Second, the diminished activation of G-protein-coupled receptors—specifically GPR41 (FFAR3) and GPR43 (FFAR2)—drastically reduces the secretion of glucagon-like peptide-1 (GLP-1) and peptide YY, thereby impairing insulin sensitivity and appetite regulation (Zhang et al., 2022). Third, SCFAs are structurally vital for maintaining the intestinal epithelial barrier, as their absence downregulates tight junction proteins such as ZO-1 and occludin, thereby enabling the unchecked translocation of LPS and other endotoxins (Roy et al., 2025). The resulting “leaky gut” accelerates systemic endotoxemia, vigorously driving IR and hyperandrogenism (Qian et al., 2026). Preclinical models confirm that replenishing SCFA pools via resistant starch or arabinoxylan supplementation successfully reverses these defects, re-establishing estrous cyclicity and metabolic balance (Liang et al., 2025).

Nevertheless, the “double-edged sword” nature of SCFAs becomes highly relevant within the context of early carcinogenesis. While generally protective against malignancies like colorectal cancer—by enforcing apoptosis in transformed cells and sustaining epithelial health—their biological effects vary according to local concentration, tissue context, metabolic state, and cellular lineage (Ravat et al., 2024). Through its HDAC inhibitory activity, butyrate extensively alters the epigenetic landscape regulating the cell cycle and apoptosis. If localized signaling networks are already disrupted, this epigenetic remodeling may have context-dependent effects on cell survival and inflammatory signaling, particularly in metabolically altered tissues (Zhang et al., 2022). For instance, while butyrate definitively induces apoptosis in established colon cancer cell lines, it can paradoxically serve as an oxidative energy source for normal colonocytes, and its localized immune modulation can oscillate between immunostimulatory and profoundly immunosuppressive depending on the local milieu (Duan et al., 2021).

Applied to PCOS—a condition carrying an elevated risk for endometrial, and potentially ovarian, malignancies—the transition from a fiber-rich to a fiber-depleted dietary pattern may have stage-dependent effects on metabolic inflammation and epithelial vulnerability. The initial loss of SCFA-mediated anti-inflammatory protection actively promotes early pre-neoplastic changes. However, aggressively reintroducing SCFAs into an already established, metabolically deranged microenvironment might yield unpredictable consequences, potentially suppressing nascent lesions or, conversely, providing metabolic fuel for specific transformed phenotypes. Thus, the maturation of the precancerous niche is dictated by this complex interplay between the loss of primary SCFA protection and the need to contextualize SCFA signaling according to timing, tissue state, and metabolic background (Chen et al., 2026). While targeted dietary interventions to elevate SCFAs remain highly beneficial for resolving the metabolic and inflammatory axes of PCOS, their long-term implications regarding oncogenic risk mandate rigorous evaluation (Zhao et al., 2026). This dual nature of SCFAs underscores the absolute necessity for precision nutrition—strategies meticulously tailored to optimize metabolite profiles for metabolic rescue without inadvertently fueling pathological progression.

## 6 Intervention strategies: targeting the gut-adipose-ovary axis

**Figure 2:**
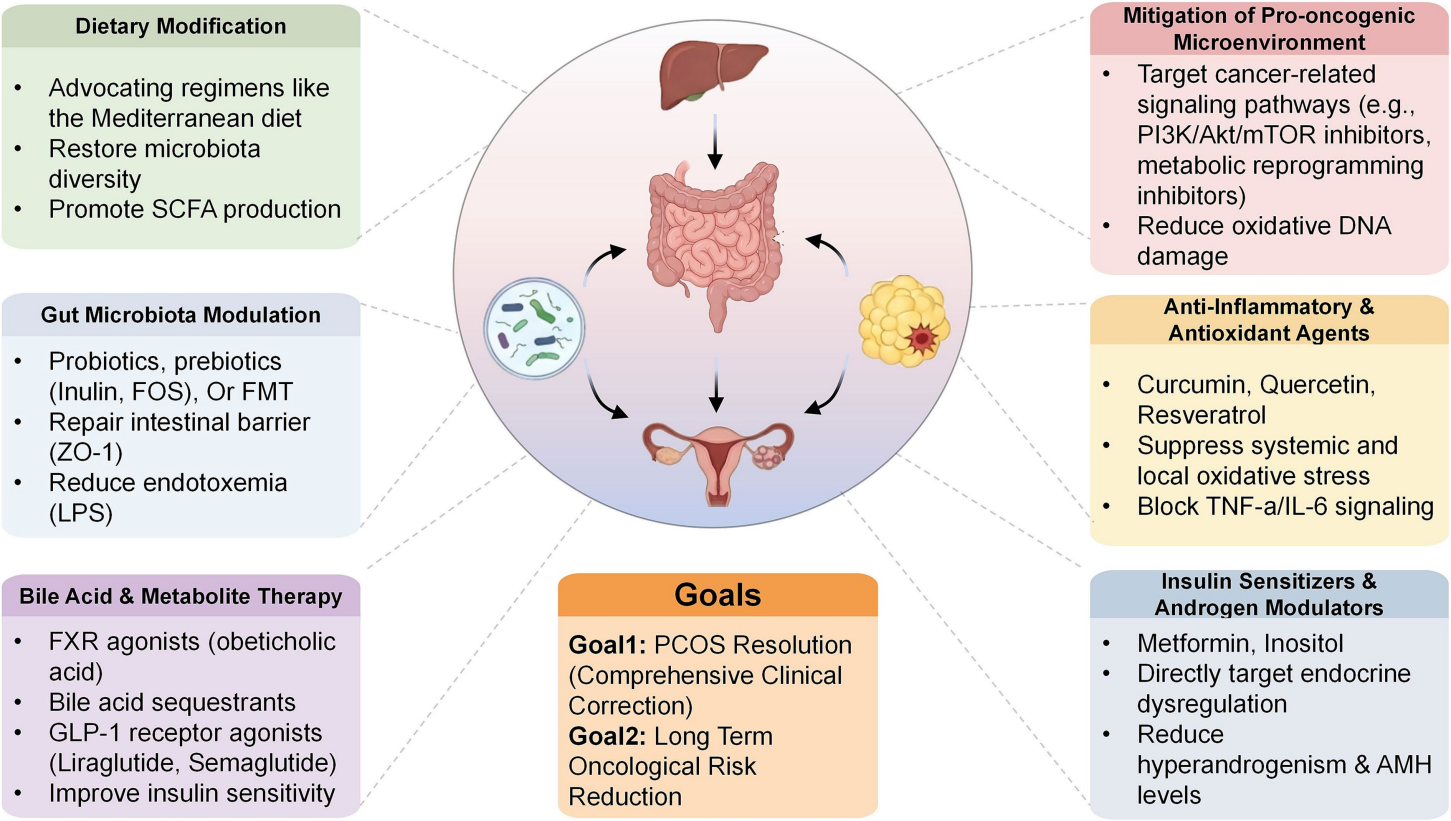
Multi-dimensional intervention strategies targeting the remodeled gut–adipose–ovary axis in PCOS. This diagram systematically summarizes six core therapeutic intervention zones, along with their specific molecular targets and mechanisms, aimed at reversing the pathological remodeling of the “gut-adipose-ovary axis.” Through multi-dimensional synergistic interventions, the goal is to break the vicious cycle from PCOS metabolic dysregulation to preneoplastic niche formation, achieving the dual clinical objectives of Goal 1: PCOS Resolution (comprehensive clinical correction) and Goal 2: Long-term Oncological Risk Reduction. (1) Dietary Modification: Advocating regimens like the Mediterranean diet to restore microbiota diversity and promote SCFA production. (2) Gut Microbiota Modulation: Repairing the intestinal barrier (ZO-1) and reducing endotoxemia (LPS) via probiotics, prebiotics (Inulin, FOS), or fecal microbiota transplantation (FMT). (3) Bile Acid and Metabolite Therapy: Utilizing FXR agonists (obeticholic acid), bile acid sequestrants, and GLP-1 receptor agonists (Liraglutide, Semaglutide) to improve insulin sensitivity. (4) Insulin Sensitizers and Androgen Modulators: Such as metformin and inositol, directly targeting endocrine dysregulation and reducing hyperandrogenism and AMH levels. (5) Anti-inflammatory and Antioxidant Agents: Using agents like Curcumin, Quercetin, and Resveratrol to suppress systemic and local oxidative stress and block TNF-α/IL-6 signaling. (6) Mitigation of Pro-oncogenic Microenvironment: Target cancer-related signaling pathways (e.g., PI3K/Akt/mTOR inhibitors, metabolic reprogramming inhibitors) to reduce oxidative DNA damage. These strategies collectively promote the restoration of the axis toward healthy homeostasis.

Given the systemic nature of PCOS pathophysiology, reversing the pathological remodeling of the gut-adipose-ovary axis requires a multi-dimensional therapeutic framework. Rather than solely palliating isolated reproductive or metabolic symptoms, clinical interventions must target the root physiological drivers to break this vicious cycle. By integrating targeted nutritional protocols, microbial ecosystem modulation, and advanced pharmacological agents, this comprehensive clinical pathway aims to improve metabolic-endocrine dysfunction and attenuate inflammatory and metabolic conditions that may support cancer-related risk in susceptible patients. As visually synthesized in Figure 2, therapeutic strategies can be organized according to several major intervention domains, including dietary modification, microbiota modulation, metabolite signaling, insulin sensitization, adipokine regulation, and anti-inflammatory or antioxidant approaches. Complementing this visual guide, Table 2 provides a structured summary of nutritional, microbial, and pharmacological strategies, highlighting their mechanistic targets and potential clinical relevance for restoring axis homeostasis and supporting long-term risk reduction.

**Table 2:** Multi-dimensional intervention strategies targeting the gut-adipose-ovary axis.

Intervention category	Specific approaches/agents	Mechanistic targets and reported/potential effects	References
Nutritional modulation	Mediterranean diet (MedDiet), low-glycemic index regimens, and supplementation with polyunsaturated fatty acids (PUFAs)	Restores microbial diversity, fortifies the intestinal epithelial barrier via enhanced SCFA production, and directly attenuates ovarian and systemic inflammation	Patel (2026), Li S. et al. (2025), Zhang H. et al. (2023), Di Lorenzo et al. (2023)
Microbiome-targeted therapies	Prebiotics (e.g., inulin, FOS) and probiotics (*Lactobacillus* and *Bifidobacterium* strains); novel synbiotic formulations (e.g., *Lactiplantibacillus pentosus* GSSK2)	Optimizes BMI and lipid profiles, actively reverses dysbiosis, mitigating metabolic endotoxemia, and suppresses systemic inflammation and may attenuate cancer-related microenvironmental risk	Liao M. et al. (2021), He et al. (2026), Khanna et al. (2021)
Pharmacological agents	Metformin, Glucagon-like peptide-1 receptor agonists (GLP-1 RAs), Toll-like receptor 4 (TLR4) inhibitors, and novel adiponectin receptor agonists	Enriches SCFA-producing taxa (e.g., *A. muciniphila*), interrupts endotoxemia-driven TLR4 inflammatory cascades, and dismantles the lipotoxic environment supporting the pre-neoplastic niche	Bashir et al. (2022), Wiggins et al. (2025), Madsbad and Holst (2023)
Advanced/metabolite interventions	Fecal microbiota transplantation (FMT) and protective bile acid (BA) supplementation (e.g., tauroursodeoxycholic acid [TUDCA])	Fundamentally repairs intestinal barrier function, mitigates cellular endoplasmic reticulum stress, restores oocyte quality, and normalizes the inflammatory milieu	Freitas et al. (2023), Di Lorenzo et al. (2023), Huang et al. (2024)

### 6.1 Nutritional, prebiotic, and probiotic interventions

Dietary interventions, strategically paired with prebiotics, probiotics, and synbiotics, represent the foundational approach for restoring gut-adipose-ovary axis homeostasis. The MedDiet—characterized by a high intake of complex carbohydrates, fiber, and unsaturated fats—has emerged as a particularly efficacious nutritional strategy. It directly ameliorates core PCOS features, including IR, hyperandrogenism, and chronic inflammation, largely by remodeling the intestinal microbial architecture (Di Lorenzo et al., 2023). These low-glycemic, fiber-rich diets promote the proliferation of commensal taxa that ferment dietary fibers into SCFAs. As critical signaling molecules, SCFAs fortify the intestinal epithelial barrier, curtail systemic LPS translocation, and exert profound anti-inflammatory and insulin-sensitizing effects, thereby directly neutralizing the core metabolic derangements of PCOS.

Parallel to whole-diet approaches, targeted microbiome manipulation via prebiotics [such as inulin and fructooligosaccharides (FOS)] and probiotics (specifically *Lactobacillus* and *Bifidobacterium* strains) offers synergistic benefits (Di Lorenzo et al., 2023; Liao M. et al., 2021). Clinical and preclinical data confirm that these interventions significantly optimize body mass index (BMI) and lipid profiles, actively reversing the dysbiosis characteristic of PCOS (He et al., 2026). Notably, maternal probiotic supplementation during pregnancy can even induce favorable microbial colonization in the infant gut, highlighting a potential avenue for intergenerational metabolic modulation (Zaidi et al., 2021). By re-establishing gut homeostasis, these nutritional strategies actively attenuate the chronic low-grade inflammation requisite for precancerous niche formation. The consumption of functional foods containing probiotics and prebiotics provides an optimal target for the development of novel strategies to combat obesity, a primary risk factor for PCOS progression (Hijova, 2021). Recognizing that baseline microbial architecture dictates individual therapeutic responses, the integration of precision nutrition is rapidly evolving to optimize these benefits for specific patient profiles (Gibbons et al., 2022; Odriozola et al., 2024). The efficacy of such microbiome-targeted interventions is further validated by their success in managing gestational diabetes mellitus (GDM)—a condition sharing distinct pathophysiological overlaps with PCOS (Lin et al., 2026). Furthermore, novel synbiotic formulations, such as *Lactiplantibacillus pentosus* GSSK2 combined with isomalto-oligosaccharides, have demonstrated robust protective efficacy against experimental metabolic syndrome by mitigating dysbiosis, enhancing glucose clearance, and suppressing systemic inflammation (Khanna et al., 2021). Collectively, these interventions form a potent first-line defense against the progression from metabolic dysfunction to ovarian oncogenesis.

### 6.2 Pharmacological agents and novel therapeutic targets

Beyond lifestyle modifications, pharmacological agents and emerging therapeutic targets are being aggressively investigated to directly disrupt the pathogenic gut-adipose-ovary axis. Insulin sensitizers, primarily metformin, remain the pharmacological cornerstone of PCOS management. While classically understood to suppress hepatic gluconeogenesis, emerging evidence indicates that metformin’s efficacy is significantly mediated by gut microbiota modulation. Specifically, it enriches SCFA-producing taxa and *Akkermansia muciniphila*—a mucin-degrading bacterium strongly correlated with metabolic health (Bashir et al., 2022). This microbial remodeling highlights the gut ecosystem as a critical interface through which classical metabolic drugs exert their systemic benefits.

Simultaneously, novel therapeutic pipelines are targeting key molecular bridges, notably TLR4. Because TLR4 operates as the primary immune sensor for translocated LPS, its chronic activation drives the inflammatory and insulin-resistant cascades central to ovarian dysfunction. Pharmacological blockade of TLR4 therefore represents a highly promising strategy to interrupt this endotoxemia-driven pathology (Bashir et al., 2022; Wiggins et al., 2025). Within adipose tissue, correcting the adipokine imbalance is equally critical. Because circulating adiponectin levels are pathologically suppressed in obesity and PCOS, novel adiponectin receptor agonists are being developed to restore its potent anti-inflammatory and insulin-sensitizing properties (Polito et al., 2020). These approaches aim to directly mitigate the pro-inflammatory and insulin-resistant state, thereby dismantling the lipotoxic environment supporting the pre-neoplastic niche (Bashir et al., 2022). Direct antagonism of elevated pro-inflammatory cytokines, such as IL-6, provides another targeted avenue to stall the progression of the disease.

The incretin system serves as another potent therapeutic node. Glucagon-like peptide-1 receptor agonists (GLP-1 RAs), including liraglutide and semaglutide, exert substantial weight-loss and glycemic-control effects via the gut-brain axis while favorably modifying the microbial landscape (Madsbad and Holst, 2023). For a more profound structural remodeling of the gut ecosystem, FMT has demonstrated remarkable preclinical potential. In PCOS models, FMT effectively reverses key syndromic features by fundamentally repairing intestinal barrier function, eradicating metabolic endotoxemia, and normalizing the inflammatory milieu (Di Lorenzo et al., 2023; Huang et al., 2024). However, translating FMT to human PCOS cohorts necessitates rigorous long-term studies to standardize donor selection and understand complex host-microbiome interactions. Finally, intestinal cytokines like IL-22 are under investigation for their ability to reinforce epithelial integrity and antimicrobial defenses (Han et al., 2022; Melchior et al., 2024). Collectively, this advanced pharmacological armamentarium aims not merely to palliate symptoms, but to fundamentally reset the metabolic-inflammatory landscape, thereby averting the long-term oncogenic sequelae driven by axis dysregulation.

## 7 Research challenges and future directions

### 7.1 The complexity of mechanistic research and model limitations

The intricate interplay within the gut-adipose-ovary axis presents a formidable challenge for mechanistic research in PCOS, primarily due to its multi-organ, multi-system nature. This axis integrates signals from the gut microbiome, adipose tissue, the HPO axis, and the immune system, forging a bidirectional communication network where perturbations in a single compartment precipitate cascading, often counterintuitive, systemic effects. While gut dysbiosis triggers systemic inflammation and IR that impair ovarian steroidogenesis and follicular development, ovarian hyperandrogenism reciprocally compromises adipose tissue function and the integrity of the intestinal barrier (Liao B. et al., 2021). This complexity is further amplified by the pronounced clinical heterogeneity of PCOS, where distinct phenotypic presentations (e.g., hyperandrogenic versus normoandrogenic, lean versus obese profiles) are likely underpinned by divergent mechanistic pathways operating within this axis (Cussen et al., 2025).

A critical bottleneck in current research is the profound reliance on animal models, which, despite their foundational utility, possess inherent translational constraints. While prenatally androgenized (PNA) mouse models exhibit high transcriptomic fidelity to human PCOS (Ren et al., 2022) and remain indispensable for investigating the developmental origins of the syndrome (Liu et al., 2024; Stener-Victorin, 2022), they cannot comprehensively recapitulate the chronic, lifelong, and polygenic etiology of the human disease. Letrozole- or testosterone propionate-induced rodent models effectively simulate specific reproductive and metabolic aberrations—such as estrous cycle disruption, hyperandrogenism, and IR—yet their capacity to model progressive, decades-long pathophysiological shifts, such as the maturation of a precancerous niche, is severely restricted (Kulkarni et al., 2025; Ren et al., 2024). Moreover, the majority of clinical evidence stems from cross-sectional population studies. While these investigations successfully correlate gut microbiota composition and metabolite profiles with PCOS metabolic markers, they inherently fail to establish causality or map the dynamic temporal evolution of the axis toward malignant transformation (Zhou et al., 2023). There is a critical deficit of prospective longitudinal cohorts tracking women with PCOS from initial diagnosis through the potential emergence of ovarian pathologies, such as borderline tumors or epithelial ovarian carcinomas. Such study designs are imperative to determine whether specific axis dysregulations—namely persistent low-grade inflammation or aberrant bile acid metabolism—hold definitive predictive value for future carcinogenesis.

Bridging these translational gaps necessitates the adoption of more sophisticated, human-relevant preclinical models. Advancements in organoid technology, particularly the engineering of co-culture systems integrating intestinal, adipose, and ovarian organoids, offer a promising platform for interrogating cell–cell and organ-organ crosstalk within a highly controlled, human-specific microenvironment (Xiao and Zhou, 2020). Concurrently, humanized animal models—such as mice engrafted with human immune components or gut microbiota—can more faithfully replicate the immuno-metabolic interactions foundational to the axis (Cao et al., 2022). Ultimately, deconstructing the complexity of the gut-adipose-ovary axis and its contribution to PCOS progression requires a highly integrated approach, merging advanced preclinical modeling with long-term, multi-omics human cohort studies.

### 7.2 Perspectives on translational medicine and precision prevention

Translating the mechanistic insights of the gut-adipose-ovary axis into clinical utility fundamentally depends on identifying robust biomarkers for risk stratification and engineering targeted preventive interventions. The paramount objective is to pinpoint which patients with PCOS harbor the highest susceptibility for progressing to ovarian precancerous lesions—such as endometrioid or clear cell carcinomas evolving from an inflammatory, hyperestrogenic milieu, or high-grade serous carcinomas with potential extraovarian origins.

Future research must prioritize the definition of a highly specific “risk signature.” This composite metric would integrate specific gut microbial taxa (e.g., the depletion of butyrate-producing populations or the enrichment of pro-inflammatory *Lactobacillus* or Proteobacteria strains) (Luginbuhl and South, 2022; Zhou et al., 2023), systemic metabolite profiles (including elevated 11-oxygenated androgens, specific bile acids, or altered SCFAs) (Cussen et al., 2025; Zhang S. et al., 2025), and circulating inflammatory mediators (e.g., IL-6, TNF-α, or high-sensitivity C-reactive protein) to reliably forecast the transition from benign metabolic dysfunction to a malignant phenotype (Liu et al., 2022; Marchiori et al., 2024). Formulating and prospectively validating a predictive algorithm based on this multidimensional signature would catalyze a paradigm shift from empirical, “one-size-fits-all” symptom management to a personalized, risk-stratified framework for oncological prevention. Operationally, this entails transitioning patient care from the mere palliation of hirsutism or oligo-anovulation toward active interventions designed to preempt malignant transformation.

A primary conduit for this preventive strategy is the targeted modulation of the gut-adipose-ovary axis via personalized nutritional and microbiome-directed therapeutics. Because dietary patterns profoundly dictate gut microbial architecture, individualized nutritional regimens that cultivate a diverse, anti-inflammatory microbiota stand as the cornerstone of early prophylaxis. Such protocols could leverage specific prebiotics or probiotics—such as *Limosilactobacillus reuteri*, which has demonstrated efficacy in modulating the gut-ovary axis in preclinical models (Geng et al., 2026)—or employ “postbiotic” supplementation with essential metabolites to fortify the intestinal-immune barrier. Strategically pairing microbiome modulation with established metabolic sensitizers (like metformin) or bioactive phytochemicals (such as quercetin (Su et al., 2024) or laurolitsine (Zhang Y. et al., 2025), both proven to ameliorate metabolic and ovarian parameters in PCOS models) could yield highly synergistic “microbiome-metabolic” therapies. This combinatorial approach actively dismantles the self-perpetuating triad of hyperandrogenism, IR, and inflammation that anchors the precancerous niche, achieving a profound “stage shift” where the clinical endpoint elevates from symptom suppression to the long-term mitigation of chronic metabolic disease and cancer-related risk.

Realizing this vision necessitates a rigorous systems biology framework. The massive datasets generated from multi-omics profiling—spanning metagenomics, metatranscriptomics, metabolomics, and proteomics—in PCOS cohorts across various disease stages must be seamlessly integrated via advanced computational modeling (Xu et al., 2023). By constructing dynamic network topologies of the gut-adipose-ovary axis, researchers can isolate critical mechanistic nodes, such as rate-limiting metabolic enzymes or specific signaling receptors, that drive pathological progression. Once identified, these nodes become highly viable targets for novel pharmacotherapy (e.g., small molecule inhibitors or subtype-specific GnRH antagonists) (Tezuka et al., 2023) or act as high-fidelity diagnostic biomarkers. Ultimately, this comprehensive, data-driven strategy will establish the systems-level evidence required to pivot from reactive clinical management to proactive, precision-based prevention for PCOS and its associated long-term sequelae.

## 8 Conclusion

The conceptual framework of the nutrition-mediated gut-adipose-ovary axis drives a critical paradigm shift in our understanding of PCOS, redefining it from a localized reproductive disorder to a systemic metabolic-endocrine continuum. As synthesized in this review, aberrant nutritional intake acts as the primary environmental catalyst, dismantling microbial homeostasis and provoking adipose tissue inflammation. This pathogenic cascade collectively enforces the clinical hallmarks of PCOS—hyperandrogenism, IR, and chronic low-grade inflammation. Crucially, this is a dynamic, self-reinforcing network that progressively reshapes the ovarian microenvironment. By sustaining proliferative signaling, inducing oxidative DNA damage, forcing metabolic reprogramming, and fostering an immunosuppressive milieu, these cumulative insults construct a veritable precancerous ecological niche. Consequently, the elevated risk of certain gynecological malignancies, particularly endometrial cancer, should be considered as a possible long-term consequence of unresolved metabolic-endocrine dysregulation in susceptible subgroups of women with PCOS, rather than as an inevitable outcome of the syndrome.

Translating this framework into clinical practice offers an unprecedented opportunity for holistic intervention, yet it demands rigorous precision. While broad nutritional strategies (e.g., the MedDiet), microbiome modulators (prebiotics/probiotics), and pharmacological agents (such as metformin and GLP-1 RAs) can concurrently intercept multiple nodes of this axis, a “one-size-fits-all” approach is fundamentally inadequate. The inherent clinical heterogeneity of PCOS endotypes—whether predominantly driven by hyperandrogenemia, profound IR, or lipotoxic inflammation—requires nuanced, mechanism-specific management. Therefore, future clinical algorithms must stratify patients based on their dominant axis disturbances. A formidable challenge lies in disentangling which specific interventions effectively arrest precancerous progression, as opposed to those that merely palliate the superficial metabolic syndrome. Overgeneralization risks deploying counterproductive therapies in highly individualized microenvironments.

To transition this axis concept from a compelling molecular hypothesis to an actionable clinical tool, a disciplined, multi-omics research agenda is imperative. Future investigations must move beyond mere taxonomic profiling of gut microbiota abundance toward integrated functional multi-omics approaches—particularly metabolomics and epigenomics—to capture the intricate metabolic and regulatory depth of these systemic interactions. Integrating such datasets will yield the robust biomarker signatures necessary for dynamic risk prediction. Furthermore, randomized controlled trials must advance beyond metabolic markers to prioritize cancer-relevant endpoints, such as the regression of endometrial hyperplasia or the suppression of localized oncogenic signals. Ultimately, by rigorously dissecting the ecological, metabolic, and oncological mechanisms governing this axis, we can pioneer proactive, precision-based preventive strategies. This paradigm shift will empower clinicians to fundamentally interrupt the disease continuum, mitigating the long-term oncological burden and transforming lifelong health outcomes for women affected by this complex syndrome.

## Glossary

AMH: Anti-Müllerian hormone
BAT: Brown adipose tissue
BMI: Body mass index
FMT: Fecal microbiota transplantation
FOS: Fructooligosaccharides
FXR: Farnesoid X receptor
GDM: Gestational diabetes mellitus
GLP-1: Glucagon-like peptide-1
GLP-1 RAs: Glucagon-like peptide-1 receptor agonists
gWAT: Gonadal white adipose tissue
HDAC: Histone deacetylase
HFD: High-fat diet
HOMA-IR: Homeostatic model assessment for insulin resistance
HPO Axis: Hypothalamic–pituitary-ovarian axis
IGF-1: Insulin-like growth factor 1
IL-1β: Interleukin-1β
IL-22: Interleukin-22
IL-6: Interleukin-6
IR: Insulin resistance
KD: Ketogenic diet
LPS: Lipopolysaccharides
MDSCs: Myeloid-derived suppressor cells
MedDiet: Mediterranean diet
MMP3: Matrix metalloproteinase-3
NAFLD: Nonalcoholic fatty liver disease
NF-κB: Nuclear factor kappa-light-chain-enhancer of activated B cells
PCOS: Polycystic ovary syndrome
RNS: Reactive nitrogen species
ROS: Reactive oxygen species
SCFAs: Short-chain fatty acids
SHBG: Sex hormone-binding globulin
T2DM: Type 2 diabetes mellitus
TAMs: Tumor-associated macrophages
TGR5: G protein-coupled bile acid receptor 1
TLR4: Toll-like receptor 4
TNF-α: Tumor necrosis factor-alpha
Tregs: Regulatory T cells
VAT: Visceral adipose tissue
WAT: White adipose tissue
ZO-1: Zonula occludens-1
